# A protocol for a scoping review of equity measurement in mental health care for children and youth

**DOI:** 10.1186/s13643-020-01495-3

**Published:** 2020-10-07

**Authors:** William Gardner, Stuart G. Nicholls, Graham J. Reid, Brian Hutton, Candyce Hamel, Lindsey Sikora, Mina Salamatmanesh, Laura Duncan, Katholiki Georgiades, Jason Gilliland

**Affiliations:** 1 School of Epidemiology, Public Health and Preventive Medicine, University of Ottawa, Ottawa, Ontario Canada; 2 CHEO Research Institute, 401 Smyth Rd, Ottawa, Ontario Canada; 3 Ottawa Hospital Research Institute, Ottawa, Ontario Canada; 4 Departments of Psychology, Family Medicine & Paediatrics, Children’s Health Research Institute, University of Western Ontario, London, Ontario Canada; 5 Library, Faculty of Health Sciences, University of Ottawa, Ottawa, Ontario Canada; 6 Offord Centre for Child Studies, McMaster University, Hamilton, Ontario Canada; 7 Departments of Geography, Paediatrics, Health Studies, Epidemiology & Biostatistics, Children’s Health Research Institute, University of Western Ontario, London, Ontario Canada

**Keywords:** Mental health, Child, Health services, Healthcare disparities, Review

## Abstract

**Background:**

Mental health (MH) problems are among the most important causes of morbidity and mortality for children and youth. Problems of lack of equity in child and youth MH services (CYMHS)—including, but not limited to, problems in inaccessibility and quality of services—are widespread. Characterizing the nature of equity in CYMHS is an ongoing challenge because the field lacks a consistent approach to conceptualizing equity. We will conduct a scoping review of how equity in MH services for children and youth has been defined, operationalized, and measured. Our objectives are to discover: (1) What conceptual definitions of equity are used by observational studies of CYMHS?; (2) What service characteristics of CYMHS care do indices of equity cover?; (3) What population dimensions have been used to operationalize equity?; (4) What statistical constructs have been used in indices that measure CYMHS equity?; and (5) What were the numerical values of those indices?

**Methods:**

The following databases will be searched: Medline, Embase, PsycINFO, Cochrane Controlled Register of Trials, CINAHL, EconLit, and Sociological Abstracts. Searches will be conducted from the date of inception to the end of the last full calendar year (December 2019). Studies will be included if they include an evaluation of a mental health service for children or youth (defined as those under 19 years of age) and which quantify variation in some aspect of child or youth mental health services (e.g., accessibility, volume, duration, or quality) as a function of socio-demographic and/or geographic variables. Study selection will occur over two stages. Stage one will select articles based on title and abstract using the liberal-accelerated method. Stage two will review the full texts of selected titles. Two reviewers will work independently on full-text reviewing, with each study screened twice using pre-specified eligibility criteria. One reviewer will chart study characteristics and indices to be verified by a second reviewer. Reviewers will resolve full-text screening and data extraction disagreements through discussion. Synthesis of the collected data will focus on compiling and mapping the types and characteristics of the indices used to evaluate MH services equity.

**Discussion:**

The planned, systematic scoping review will survey the literature regarding how equity in MH services for children and youth has been operationalized and help inform future studies of equity in CYMHS.

**Systematic review registration:**

Open Science Foundation ID SYSR-D-19-00371, https://osf.io/58srv/.

## Background

### Rationale

Mental health (MH) problems are major sources of morbidity and significantly impair children’s development. The prevalence of mental health disorders in children and adolescents has been estimated to be between 15% [1] and 20% [2] globally, with studies indicating that median age of onset for any disorder being 14 years. Higher rates of child and youth mental health (CYMH) problems tend to occur in poorer countries, although the country was not a substantive factor in prevalence estimates within multivariate models [3]. Within North America, mental health disorders affect nearly one person in 5 [3] and 13-20% of children living in the USA and Canada experience a mental disorder [1, 4–6]. Child and youth mental disorders are a well-documented source of morbidity and mortality during childhood and youth, with lasting effects throughout adulthood [7–15]. These effects include but are not limited to difficulties in the workplace [16], poor parenting skills [17], adult imprisonment [18], and many adult mental disorders, including anxiety disorders [19], substance use disorder [20, 21], depression [22, 23], bipolar disorder and schizophrenia [24, 25], and post-traumatic stress disorder [26, 27]. Unfortunately, and contrary to principles of universal health care, many jurisdictions have inequities in the accessibility and quality of mental health services [28–37]. Moreover, problems with equitable access to child and youth mental health services (CYMHS) are an international issue [38]. Gaps in systems of care, multiple barriers to accessing services, gaps in policies specific to CYMHS, and issues with training and financing are common worldwide [39], with these issues being even more pronounced in low- and middle-income countries.

The current project initiates a program of research on equity in child and youth mental health services (CYMHS). While the definition of equity is contested, it is commonly described in terms of its inverse, inequity. In their review of interventions to promote mental well-being in children and youth, Welsh et al. define health inequities as “differences in health status between population groups that are socially produced, systematic in their unequal distribution, avoidable and unfair” [28]. This may be operationalized through an “equity lens” [40] involving the formal assessment of whether there is a differential impact of an intervention according to some factors that are deemed socially stratifying [28, 40]. Several groups have sought to develop a formal list of categories upon which to evaluate equity. One such example is the PROGRESS-Plus framework, which organizes equity into the domains of the following: the place of residence, race/ethnicity/culture/language, occupation, gender/sex, religion, education, socioeconomic status, and social capital [41]. This framework has now been applied to several reporting guidelines to create extensions to the CONSORT reporting guideline for clinical trials [42] and the development of the PRISMA-Equity extension [43–45].

While several reviews exist in relation to the mental health of children and youth and provide support for the notion that equity is an important aspect of CYMHS to consider, these reviews tend to focus on a specific subgroup of the population (e.g., indigenous children) [45] or type of study (e.g., longitudinal cohorts) [46]. Our review differs in important ways. First, the target population is much broader and more comprehensive. Second, the focus is on mental health services rather than the prevalence of psychopathology.

Clarifying how equity has been conceptualized and measured is an important first step in our research program. This review will scope approaches to measuring equity in studies on MH service delivery for children and youth to identify crucial gaps and inconsistencies in the area. We chose to conduct a scoping review because of the diverse body of literature on equity in CYMHS and the lack of prior data about how equity has been operationalized [37].

### Objectives

The primary goal of this review is to survey the indices of equity used in the literature, where an “index” is a quantitative measure of equity (or inequity) that pertains to perceived unfair distribution of care in the delivery of CYMH services. For each index, we will identify the following:

1. What *conceptual definitions* of equity—if any—are used in studies of CYMHS? Although there are many empirical studies of equity, few of these studies define what equity/inequity is, or how fairness/unfairness is assessed.

2. What *service characteristic(s)* of CYMHS care does the index cover? Examples of service characteristics include effectiveness, quality, and accessibility.

3. What *population dimensions* have been used to operationalize equity? Inequity concerns how something valuable—in this case, the service characteristic—differs across a population. The members of a population, however, vary along many axes or dimensions: for example, age, gender, or the region where people live. A service equity index will isolate one or more dimensions and measure variation in the service characteristic along those dimensions. For example, in a study of gender equity in income, income is the thing of value, and gender is the population dimension along which it varies.

4. What *statistical construct* is used in the equity index? Variation is measured using statistics; therefore, equity indices are statistical constructs.

5. What is the *numerical value* of the index?

## Methods/Design

A scoping review will be performed using methods from Arksey and O’Malley [47] with further refinement from guidance by the Joanna Briggs Institute [48]. This study protocol has been posted to the Open Science Foundation (https://osf.io/, ID SYSR-D-19-00371). The protocol is written in accordance with the Preferred Reporting Items for Systematic Reviews and Meta-Analyses Protocols (PRISMA-P) statement [49]. Any protocol modifications made during the conduct of the review will be described in the publication of the final report.

### Data sources and search for studies

An experienced information specialist developed preliminary search strategies in consultation with the review team (Additional file 1). The search strategy draws upon existing search strings developed by the Cochrane Equity group [50] with terms previously used in systematic reviews of MH care and coverage [51, 52]. We will search the following databases: Medline (via Ovid), Embase (via Ovid), PsycINFO (via Ovid), Cochrane Controlled Register of Trials (via Ovid), CINAHL (via EBSCOHost), EconLit (via Proquest), and Sociological Abstracts (via Proquest). The syntax will be adjusted according to the needs of each database. All databases will be searched from their date of inception to the end of the last full calendar year (December 2019), and references will be imported into Endnote x7 (Clarivate Analytics). The search strategy will not restrict citations by language; however, for feasibility, only studies written in English and French will be included at the screening level. Articles in other languages will be excluded, and a list of the potentially relevant studies will be provided in a supplement of the final report for interested readers.

The search will be subject to independent peer review by another experienced librarian using the Peer Review of Electronic Search Strategies (PRESS) checklist [53]. The grey literature will be searched using keywords from the search strategy. Specifically, we will examine sites from Canada, including the Mental Health Commission of Canada, the Canadian Paediatric Society, the Centre for Addiction and Mental Health, the Canadian Psychological Association, the College of Family Physicians of Canada, the Canadian Academy of Child and Adolescent Psychiatrists, the Canadian Nurses Association, and the Canadian Psychiatric Association. Sites from the USA will include the American College of Physicians, the American Academy of Paediatrics, the American Academy of Family Physicians, the American Academy of Child and Adolescent Psychiatrists, the American Nurses Association, the Anxiety and Depression Association of America, and the American Psychological Association. We will search for similar sites for the UK and Australia.

Bibliographies of included articles will be hand searched. We will use Scopus to find articles that cite our included articles using forward citation tracking.

### Study eligibility criteria

We set the eligibility criteria for the review according to the PCC (Population-Concept-Context) framework [48]. For this review, we are interested in studies that have operationalized equity in some way, and not those that theorize mechanisms or conceptualizations. We will include studies that meet the following criteria:

- *Population*. Studies that concern services for children (less than 19 years old) with MH concerns, including, but not limited to, externalizing (e.g., oppositional defiant disorder, conduct disorder, attention deficit hyperactivity disorder), and internalizing (e.g., anxiety, depression) problems, eating problems, and substance use. We will exclude studies of learning or developmental disabilities, except for those that include participants who are dually diagnosed with a mental health disorder.
- *Concept*. We are interested in studies that quantify variation in some aspect of mental health services (e.g., accessibility, volume, duration, or quality) as a function of socio-demographic and/or geographic variables (e.g., family income, parental educational attainment, family structure, age, sex, gender, language, race/ethnicity, communities, or distance to care) factors. Studies will be excluded if they do not consider variation specifically in relation to children and youth (e.g., report only overall rates or do not distinguish between adults and youth within a mixed sample). Study designs of interest will include observational studies (including cross-sectional studies and cohort studies). Clinical trials will be included only if we find trials whose specific aim is to enhance the equity of CYMHS. We will exclude qualitative studies (because they do not generate statistical indices), systematic review, meta-analysis, scoping reviews, literature review letters, commentaries, editorials, case reports, and case series.
- *Context.* Any mental health services setting will be considered in the review. This includes community-based CYMH agencies, mental health services delivered within the health care sector, and services within child welfare, counselling, juvenile justice, and the education system.

### Study selection

Study selection will be performed in two stages using the online systematic review software program Covidence (www.covidence.org). Following the removal of duplicate citations, screening at stage one will encompass reviewing titles and abstracts identified from the electronic searches. All articles that meet the subject matter criteria—described above will be included at this stage. Articles will be screened using the liberal accelerated method [49]. In this method, only one reviewer must identify a citation as potentially relevant for it to be moved forward to full-text screening, while two reviewers must judge a citation to be ineligible for it to be excluded. Thus, if a reviewer includes an article, that article does not need a second stage one screening.

Stage two screening will evaluate the full-text articles against the complete eligibility criteria, among those deemed potentially relevant during stage one. Each article will be screened independently by two reviewers. Disagreements among reviewers will be resolved through discussion or with a senior team member if the reviewers cannot agree.

Before each screening stage, we will calibrate the reviewers to ensure consistent application of eligibility criteria. We will continue the calibration until we reach 95% agreement between the screeners. We anticipate this will require 100 records for stage one screening of titles and abstracts and 25 records for stage two screening of full-text articles. Finally, a PRISMA flow diagram [54, 55] will be prepared to document the study selection process in the final publication. A bibliographic list of studies excluded at full-text will be provided, organized by reason for exclusion.

### Data extraction

A data extraction form will be developed and pilot-tested using a sample of 20 articles, and then revised as necessary. Items will be extracted by a single reviewer with a second reviewer verifying the content of extractions. Any disagreements will be resolved through discussion and consensus with the involvement of a third reviewer if necessary. Table 1 lists the items for data extraction. These items will be the elements of the data extraction form used by reviewers. Reviewers will capture several characteristics of the study, such as the author and date. The remaining items concern the features of the equity index and correspond to the study objectives: (a) a conceptual definition of equity (if the study includes one); (b) the characteristics of the CYMH service being studied, for example, accessibility or quality of services [56, 57]; (c) the population dimensions used to operationalize equality [58]; (d) the statistical construct used in the equity index (for example, a mean difference); and (e) the numerical value of that index.

**Table 1:** Listing of items for data extraction

*Study characteristics*	
• Author and date of publication	
• Study design (e.g., cohort, case-control)	
• Country of conduct	
• Study time period	
• Population demographics (e.g., age, ethnicity, geographic setting)	
• Number of participants	
• Study setting (e.g., outpatient, classroom)	
• Sector (e.g., specialized mental health)	
*Conceptual definition of equity*	
*Service characteristic*	
• Access to services	
• Volume of services received	
• Quality of services	
*Population dimension*	
• Sex or gender	
• Rurality	
• Language	
• Socioeconomic status	
*Statistical construct*	
• Statistics used to measure equity (e.g., difference in rates, difference in percentages, standardized mean difference, odds ratio for receipt of services, Gini curve)	
*Numerical value of the index*	

### Approach to evidence synthesis

As per Grimshaw [59], our synthesis will initially organize reports into logical categories on the basis of equity-related items considered within the studies (e.g., indices applied, socioeconomic variables considered). For qualitative data—such as conceptual definitions of equity applied—we will use a narrative synthesis approach, which will follow a strategy similar to that implemented in primary qualitative research [60]. Thus, data will be analyzed, coded, and labelled in an inductive manner using the constant comparative method. This process of coding is iterative, allowing for the revision of codes as analysis proceeds [61, 62]. For categorical and numerical data, descriptive statistics, such as frequencies, will be used to summarize the data. These statistics may be presented in tabular format or graphically (e.g., with bar charts). As scoping reviews are flexible and iterative, we may modify our approach once studies are identified, and data are charted.

## Discussion

Mental health disorders are among the most prevalent and significant health problems faced by children and adolescents. Conversely, good mental health is essential for the development of adult capabilities and the achievement of well-being. Although there are efficacious treatments for many CYMH disorders, many children and adolescents receive no treatment. Whether children and youth with CYMH disorders receive treatment is associated with social inequities and, in turn, contributes to the perpetuation of such inequities. The results of this review will help us identify gaps in the conceptualization of health equity in CYMHS, inform research designs in this domain, and help shape the development of initiatives and potential interventions on equity in CYMHS.

A particular challenge for the present review is the likely small number of studies that will explicitly reference equity considerations, despite analyses that are consistent with equity concerns. In anticipation of this, we have made use of validated search strings developed by established groups to maximize the coverage while retaining a feasible number of articles for screening. We have also engaged a number of knowledge users who have assisted us in the identification of key institutions, and which will inform our grey literature search. The inclusion of forward citation searching and grey literature searching should help to mitigate concerns regarding lack of specificity within reporting of studies with an equity concern. Further, we anticipate that studies that involve mixed populations (e.g., you and adult) may not distinguish equity considerations for children and youth compared to adult populations.

This review will inform the development and methodology of future projects on the equity of CYMHS. In particular, we hope that the results of the review will inform recommendations for standards in the reporting of mental health services equity. Progress in achieving equity in mental health services is unlikely to be achieved unless data on equity are consistently reported using standardized approaches. However, there is currently little standardization in how equity in mental health services is measured. Indices should be chosen based on how well they capture our conceptual understanding of inequity and how readily they are understood by the public. Reviewing the indices that have been used to measure equity in past research is the first step toward discussions leading toward the establishment of standard measures.

We will publish the results of this review in a health services research journal with the intent of maximizing outreach to social scientists and health services researchers pursuing research on health equity. In addition to a peer-reviewed publication, we will also draft lay summaries to post online and for distribution to key societies, patient groups, and policymakers.

## Supplementary information

**Additional file 1.** Search strategy

## Data Availability

The datasets generated and/or analyzed during the current study will be available in the Open Science Foundation repository.

## Abbreviations

*CINAH*: Cumulative Index to Nursing and Allied Health
*CYMH*: Child and youth mental health
*CYMHS*: Child and youth mental health services
*MH*: Mental health
*PRESS*: Peer Review of Electronic Search Strategies
*PRISMA*: Preferred Reporting Items for Systematic Review and Meta-Analysis
*PRISMA-ScR*: Preferred Reporting Items for Systematic Reviews and Meta-Analysis Extension for Scoping Reviews
