# Supplementary material for: A protocol for a scoping review of equity measurement in mental health care for children and youth
Source: Syst Rev. 2020 Oct 7;9:233. doi: 10.1186/s13643-020-01495-3 (PMC7542722; doi:10.1186/s13643-020-01495-3)
Supplement: Supplementary file 1 — Additional file 1. Search strategy [file 13643_2020_1495_MOESM1_ESM.docx]

Additional file 1: Search Strategy

Database: Ovid MEDLINE(R) ALL <1946 to August 07, 2019> Search Strategy:

--------------------------------------------------------------------------------

1 Health Equity/ (827)

2 Healthcare Disparities/ (14793)

3 (access* or inaccess* or inequit* or disparit* or inequalit* or equalit* or equit* or disadvantage* or discriminat* or social exclu* or social inclu* or fair or fairness or unfairness or unfair or variation* or variabilit*).tw. (1703779)

4 or/1-3 (1707731)

5 exp Mental Health Services/ (91785)

6 Psychotherapy/ or Family Therapy/ (60479)

7 adolescent psychiatry/ or child psychiatry/ (6672)

8 exp Community Mental Health Centers/ (3178)

9 Hospitals, Psychiatric/ (24883)

10 Substance Abuse Treatment Centers/ (5138)

11 ((mental* or psychiatr*) adj2 (patient* or treatment or hospital* or institut* or centre* or center* or agenc* or clinic or clinics or service or services or program*)).tw. (90266)

12 ((famil* or child* or adolescen* or teen* or youth*) adj2 (psychotherap* or psycho-therap* or therap* or counselling or counseling or intervention*)).tw. (35382)

13 or/5-12 (264365)

14 Adolescent/ or exp Child/ or exp Infant/ or Pediatrics/ or Adolescent Health Services/ or exp Child Health Services/ (3449492)

15 (child or children or childhood or infant or infants or baby or babies or toddler* or newborn or newborns or neonate or neonatal or neonates or preemie or preemies or infancy or paediatric* or pediatric* or girl or girls or boy or boys or kid or kids or teen or teens or teenage or teenager or teenagers or youngster or youngsters or youth or youths or adolescent or adolescents or adolescence or preadolescent or preadolescence or pre adolescent or pre adolescence or preschooler or school age or school aged or schoolchildren juvenile or young adult*).tw. (2280182)

16 14 or 15 (4124286)

17 4 and 13 and 16 (8772)

18 Epidemiologic studies/ (8037)

19 exp case control studies/ (1009952)

20 exp cohort studies/ (1884019)

21 Case [control.tw](http://control.tw). (117303)

22 (cohort adj (study or studies)).tw. (181641)

23 Cohort analy$.tw. (7158)

24 (Follow up adj (study or studies)).tw. (47304)

25 (observational adj (study or studies)).tw. (94761)

26 Longitudinal.tw. (226131)

27 Retrospective.tw. (481025)

28 Cross [sectional.tw](http://sectional.tw). (317448)

29 Cross-sectional studies/ (300587)

30 or/18-29 (2807151)

31 17 and 30 (2173)
